# Reviewer acknowledgement 2013

**DOI:** 10.1186/1477-9560-12-2

**Published:** 2014-01-29

**Authors:** Yukio Ozaki, Hugo ten Cate

**Affiliations:** 1 University of Yamanashi, Yamanashi, Japan; 2 Academic Hospital Maastricht, Maastricht, The Netherlands

## Abstract

**Contributing reviewers:**

The Editors of *Thrombosis Journal* would like to thank all our reviewers who have contributed to the journal in Volume 11 (2013).

## 

Walter Ageno

Italy

Tatsuya Atsumi

Japan

Fidelia Bode-Thomas

Nigeria

Annemieke Bouman

Netherlands

Sigrid Brækkan

Norway

Karen Breen

United Kingdom

Allison Burnett

United States of America

Anthonius De Boer

Netherlands

Bas de Laat

Netherlands

Pieter Eijgenraam

Netherlands

Emmanuel Favaloro

Australia

Habib Gamra

Tunisia

Esteban Gandara

Canada

Hadi Goubran

Egypt

Andreas Greinacher

Germany

Job Harenberg

Germany

Coenraad Hemker

Netherlands

Yvonne Henskens

Netherlands

Shawn Hoffman

Canada

Hisanori Horiuchi

Japan

Akira Ishiguro

Japan

Manuela Joore

Netherlands

Makoto Kaneko

Japan

Abdul Nafey Kazi

Pakistan

Marie-Claire Kleinegris

Netherlands

Tomohiro Koga

Japan

Lai Heng Lee

Singapore

Masahiro Leko

Japan

Rinske Loeffen

Netherlands

Helen Mani

Germany

Jun Mimuro

Japan

Toshiyuki Miyata

Japan

Baheramsjah Mochtar

Netherlands

Manuel Monreal

Spain

Eriko Morishita

Japan

Shosaku Nomura

Japan

Rene Oerle van

Netherlands

Martin O'Flaherty

United Kingdom

Shouichi Ohga

Japan

Tsukasa Ohmori

Japan

Paolo Prandoni

Italy

Avi Rivkind

Israel

Changgeng Ruan

China

Meyer Michel Samama

United Kingdom

Bob Siegerink

Netherlands

Sonja Sorensen

United States of America

Katsue Suzuki-Inoue

Japan

Arina ten Cate-Hoek

Netherlands

Armando Tripodi

Italy

Gabriele Vetsch

Switzerland

Alejandro Videla

Argentina

Jeanine Walenga

United States of America

Kristien Winckers

Netherlands

Yi Wu

China

Norikazu Yamada

Japan

Kazuma Yamakawa

Japan

Masako Yamazaki

Japan

Tong Yin

China

